# The impact of premature birth on dental maturation in the permanent dentition

**DOI:** 10.1007/s00784-018-2501-3

**Published:** 2018-06-08

**Authors:** Liselotte Paulsson, Sara Arvini, Niclas Bergström, Gunilla Klingberg, Christina Lindh

**Affiliations:** 1 Department of Orthodontics, Malmö University, SE-20506 Malmö, Sweden; 2 Faculty of Odontology, Malmö University, SE-20506 Malmö, Sweden

**Keywords:** Dental maturation, Tooth development, Preterm birth, Term birth, Panoramic, Radiography

## Abstract

**Objectives:**

To evaluate tooth development and calculate dental maturity score in prematurely born children and to compare the findings with full-term born children.

**Material and methods:**

Nine-year-old preterm children were selected from the Swedish Medical Birth Register. One group consisted of 36 extremely preterm children (born before week 29), and the other included 38 very preterm children (born during weeks 29 to 32). Panoramic radiography was performed on each child and the preterm children were compared with 42 full-term born children. Five observers independently assessed the tooth development stages for all teeth in the left mandible (31–37) on the panoramic radiographs according to the method described by Demirjian et al. (Hum Biol 45:211–227, 1973). Data from tooth development stages were compiled and converted into a dental maturity score for each group. Kappa values were calculated for intra- and inter-observer agreement.

**Results:**

When the different development stages for each individual tooth were compared, all observers presented a significant delay in the maturity of tooth 37 for the extremely preterm group (*p* ≤ 0.002). The extremely preterm group had a significantly lower dental maturity score than the full-term group, as assessed by each observer (*p* ≤ 0.006). Kappa values for inter-observer agreement varied between 0.31 and 0.71 depending on tooth and intra-observer agreement was between 0.16 and 1.0.

**Conclusions:**

At age 9, the extremely preterm children had a general delay in tooth development.

**Clinical relevance:**

The increased survival rate of extremely preterm babies adds a new group of children to society. Dental clinicians should be aware that the delay in tooth development could impact the timing of orthodontic diagnostics and potential treatment.

## Introduction

According to the World Health Organization, approximately 15 million children (> 10%) are born premature every year (i.e., children born before gestational week 37 or weigh below 2500 g at birth) [1]. The great improvements made in neonatal care in recent decades have led to increased survival rates for preterm and in particular extremely preterm children (born before gestational week 29) [2, 3]. Nevertheless, the shorter the gestational period, the higher the risk for morbidity and medical complications [4]. Premature birth has also been shown to affect the morphology of the craniofacial complex and the tooth size [5, 6].

A systematic review [7] has presented results from five studies [8–12] regarding tooth eruption and development among preterm and/or children born with low birth weight. Four of the studies report a delay of tooth eruption and development when based on chronological age [8–11]. Low birth weight is either the result of preterm birth or of restricted intrauterine growth; however, in some of these studies, children with low birth weight were classified as preterm even though they were not born premature. Therefore, it has been claimed that ultrasonography determines gestational age more accurately when describing the maturity of preterm infants.

It is hypothesized that the delay in tooth eruption and development is more evident in children with a lower gestational age due to the general growth delay of the rest of the body that the extremely preterm children in particular have been shown to experience [13]. It is therefore of interest to investigate the level of dental development in different groups of prematurely born children defined strictly according to their gestational age. The age group of 9 years is of specific interest, as the children are in the mixed dentition and it is when orthodontic diagnostics and treatment planning is often initiated.

Tooth development is mostly evaluated in panoramic radiographs. In previous studies, the evaluation has mainly been performed by only one observer [11, 12]. As different observers may have different visual concepts, a study on diagnostic accuracy efficacy must involve several observers. Thus, the purpose of this study was to investigate, in extremely and very preterm children at 9 years, differences in tooth development in panoramic radiographs as assessed by several observers. Another purpose was to estimate the overall dental maturity score based on development stages observed in individual teeth for each child and compare the scores between groups.

## Material and methods

### Subjects

The selection of individuals and registration of their characteristics is described in detail in the study by Paulsson et al. [14], where the purpose was to investigate the differences in malocclusion traits and orthodontic treatment needs between children born preterm and full-term. The study was approved by the Ethics Committee of the University of Lund, Sweden (Dno. LU 61-01). After permission from the Swedish Centre for Epidemiology, access to the Medical Birth Register was obtained. An epidemiologist at the Department of Epidemiology at Lund University in Sweden performed the selection from the register and created a data file of all the children born during gestational weeks 23–32 between 1992 and 1996 in the county of Scania, Sweden. The data file contained information about gestational age and birth weight for 150 extremely preterm (EPT) children born between gestational weeks 23 and 28 and 340 very preterm (VPT) children born between gestational weeks 29 and 32.

Inclusion criteria were Caucasian children aged between 8 and 10 years born at the university hospitals of Lund and Malmö and now live in the southwest region of Scania. Children with syndromes or neuromuscular disorders were excluded (7 EPT and 2 VPT).

The control group of full-term (FT) children was also recruited from the Medical Birth Register. The selection criteria were that they needed to be full-term, normal-birth-weight children who were born at the same hospital, were of the same gender and nationality, had come from the same region of Sweden, and were nearest in terms of birth month. Children born during gestational weeks 33–36 were not included in the study. The subjects who agreed to participate and fulfilled all the inclusion requirements finally consisted of 36 EPT children, 37 VPT children, and 42 FT.

### Radiographic examination

Between 2002 and 2005, panoramic radiography was performed at the Department of Oral and Maxillofacial Radiology in the Faculty of Odontology at Malmö University in Malmö, Sweden. The X-ray unit used was a Cranex 3+ Ceph (Soredex Co., Finland) with Kodak Lanex intensifying screen (Eastman Kodak Co., Rochester, NY, USA). The imaging parameters varied according to patient variation, but typically were 70 kV (constant potential) at 8 mA for 15 s. A conventional film/cassette combination was used for image capture. Normal quality criteria for panoramic radiography were used, and, wherever possible, unacceptable radiographs were repeated. The vertical magnification factor in the radiographs was 1.3 according to the manufacturer.

### Assessment of dental development stages

**Fig. 1:**
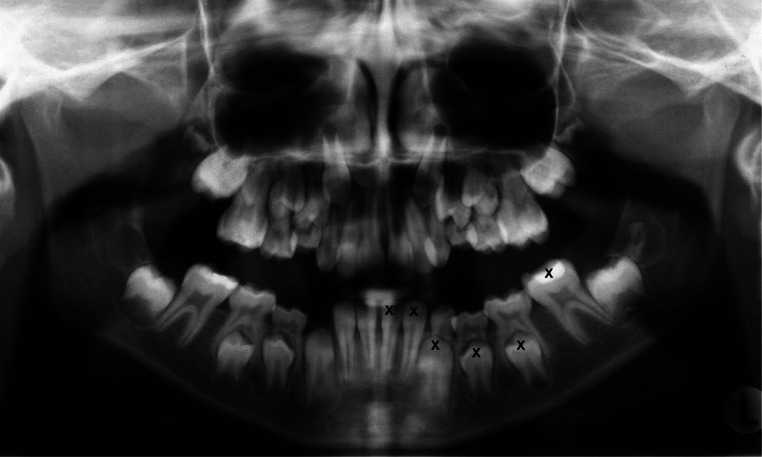
Panoramic radiograph from one of the included individuals. Teeth 31–37, which are marked with a cross, were assessed according to Demirjian et al. [15]. The criteria for the assessment of different stages varied from the beginning of calcification of crown to apical root apex completely closed (stages A–H). In Fig. 1, for example, tooth 31 was assessed as stage H and tooth 37 as stage D. If a tooth appeared to be between stages, the earlier stage was chosen

The radiographs were table-mounted, suitably masked, and studied under standardized conditions. Mattson’s binocular was available when needed. Five observers independently assessed the tooth development stages of the seven left mandibular teeth (teeth 31–37) in the panoramic radiographs according to the method described by Demirjian et al. [15] (Fig. 1). The original written descriptive criteria consisting of radiographic images and line drawings of each stage by tooth type were used to select the stages. As described in Table 1, the stages varied from the beginning of calcification of crown to apical root apex completely closed (stages A–H). If a tooth appeared to be between stages, the earlier stage was chosen. The data of the tooth development stages was compiled and converted into the dental maturity score. The reference values for the dental maturity scores were taken from the revised article from 1976 by Demirjian et al. [16]. If neither the left mandibular tooth nor the contralateral could be assessed, the patient was excluded, as the scores of all seven assessed teeth are needed to calculate the dental maturity score and avoid deviation when comparing the means between the three groups*.*

**Table 1:** Description of the different formation stages A–H according to Demirijan et al. [15]

Stage	Description
A	In both uniradicular and multiradicular teeth, a beginning of calcification is seen at the superior level of the crypt in the form of an inverted cone or cones. There is no fusion of these calcified points.
B	Fusion of the calcified points forms one or several cusps which unite to give a regularly outlined occlusal surface.
C	(a) Enamel formation is complete at the occlusal surface. Its extension and convergence towards the cervical region is seen. (b) The beginning of a dentinal deposit is seen (c) The outline of the pulp chamber has a curved shape at the occlusal border.
D	(a) The crown formation is completed down to the cementoenamel junction. (b) The superior border of the pulp chamber in the uniradicular teeth has a definite curved form, being concave towards the cervical region. The projection of the pulp horns if present, gives an outline shaped like an umbrella top. In molars, the pulp chamber has a trapezoidal form. (c) Beginning of root formation is seen in the form of a spicule.
E	Uniradicular teeth: (a) The walls of the pulp chamber now form straight lines, whose continuity is broken by the presence of the pulp horn, which is larger than in the previous stage. (b) The root length is less than the crown height. *Molars:* (a) Initial formation of the radicular bifurcation is seen in the form of either a calcified point or a semi-lunar shape. (b) The root length is still less than the crown height.
F	Uniradicular teeth: (a) The walls of the pulp chamber now form a more or less isosceles triangle. The apex ends in a funnel shape. (b) The root length is equal or greater than the crown height. Molars: (a) The calcified region of the bifurcation has developed further down from its semi-lunar stage to give the roots a more definite and distinct outline with funnel shaped endings. (b) The root length is equal to or greater than the crown height.
G	The walls of the root canal are now parallel and its apical end is still partially open (distal root in molars).
H	(a) The apical end of the root canal is completely closed. (Distal root in molars). (b) The periodontal membrane has a uniform width around the root and the apex.

Two observers were dental students, one was an orthodontist, one was an oral radiologist, and one was a specialist in pediatric dentistry. The experience of the specialists in their field of specialty ranged between 9 and 26 years (mean 17 years). The specialist in pediatric dentistry had extensive experience of using the method by Demirijan et al. [15]. The observers were unaware of to which of the groups (EPT, VPT, or FT) each panoramic radiograph belonged. Prior to the observation period, the guidelines for assessment were discussed and a calibration procedure was done in 10 panoramic radiographs of children with the same age as in the present study material. These panoramic radiographs were not included in the study. After a minimum of 2 weeks after the first assessment, all five observers assessed 23% (*n* = 27) randomly chosen panoramic radiographs a second time.

### Statistical analysis

All data were analyzed with the Statistical Package for the Social Sciences (SPSS, SPSS Inc., Chicago, IL, USA) version 21.0 software program. Numerical data and mean and standard deviations were calculated with a two-way analysis of variance with repeated measurements (ANOVA). Tukey’s post hoc test was used to test differences between groups. Chi-square analysis and Fisher’s exact test were used to determine difference between groups regarding categorical data. Differences with probabilities of less than 5% (*P* < 0.05) were considered to be statistically significant.

Kappa values were generated for intra- and inter-observer agreement [17] and categorized according to the scale suggested by Landis and Kock [18]. According to this scale, values < 0 are considered as poor agreement, 0.00–0.20 slight, 0.21–0.40 fair, 0.41–0.60 moderate, 0.61–0.80 substantial, and values > 0.81 as almost perfect agreement.

## Results

Subject characteristics’ data can be seen in Table 2

**Table 2:** Data of general characteristics for the extremely preterm (EPT), very preterm (VPT), and full-term (FT) children at birth and at investigation

	EPT group (A) *n* = 36, boys/girls 26/10	VPT group (B) *n* = 38, boys/girls 21/17	FT group (C) *n* = 42, boys/girls 23/19	
Variables	Mean (SD)	Mean (SD)	Mean (SD)	Group differences
*At birth*				
Gestational age (weeks)	26.7 (0.9)	30.7 (1.2)	39.8 (1.0)	A,B/C***, A/B***
Birth weight (g)	924 (241)	1589.2 (371)	3566.1 (474)	A,B/C***, A/B***
*At investigation*				
Age (years)	9.0 (0.6)	9.3 (0.4)	9.3 (0.5)	A/C*, A/B and B/C NS
Height (cm)	133.3 (8.1)	137.3 (6.1)	139.2 (6.7)	A/C**, A/B and B/C NS
Weight (kg)	29.5 (6.8)	32.7 (7.4)	36.5 (7.3)	A/C***, A/B and B/C NS
Head circumference (cm)	52.2 (1.5)	52.8 (1.6)	53.6 (1.5)	A/C***, B/C* and A/B NS

*NS* not significant

*p < .05; **p < .01; ***p < .001

Altogether, seven teeth in each panoramic radiograph were available for assessment, resulting in 252 teeth in total for the EPT group, 259 teeth for the VPT group, and 294 teeth for the FT group. Three of the five observers were able to assess all seven teeth of all the patients. One observer found three teeth and their contralateral to be too blurry and another observer considered one tooth to be too blurry for assessment. Given that all seven teeth are needed to calculate the total dental maturity score, the calculation for these individuals was not performed (*n* = 4).

**Fig. 2:**
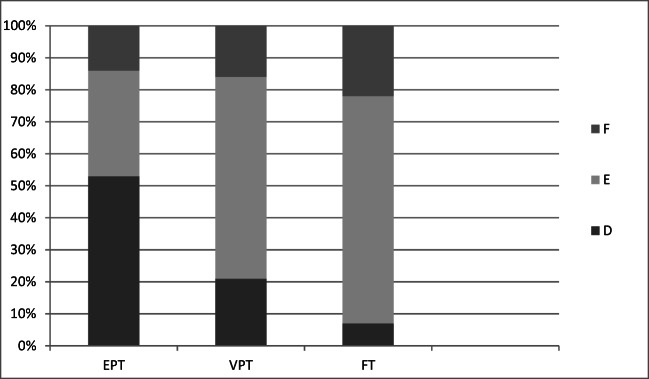
The distribution of development stages within the three groups for tooth 37 for one observer’s assessment. According to Demirjian et al. [15], the tooth development could be rated on a scale from the beginning of calcification of crown to apical root apex completely closed (see Table 1 for description of stages A–H). In all three groups, tooth 37 were classified to be in development stages D, E, or F. In stage D, the crown formation is completed down to the cemento-enamel junction. In stage E, the root length is still less than the crown height and in stage F, the root length is equal to or greater than the crown height. When comparison between groups were made, it was found that tooth 37 was significant in more undeveloped stages in both the extremely preterm group (EPT) (*p* < 0.000) and in the very preterm group (VPT) (*p* = 0.016) compared to the full-term group (FT)

According to Demirijan et al. [15], tooth development can be rated on a scale, from the beginning of calcification of crown (A) to apical root apex completely closed (H). Taking each individual tooth (31–37) into consideration, all observers assessed tooth 37 to be significant in more undeveloped stages in the EPT group compared with the FT group (*p* ≤ 0.002) (Table 3). One observer also reported that tooth 37 was significantly more undeveloped in the EPT group compared with the VPT group (*p* = 0.016). The distribution of development stages within groups for tooth 37 for this observer’s assessment is illustrated in Fig. 2. In all three groups, all five observers classified tooth 37 to be in development stages D, E, or F. In stage D, the crown formation is completed down to the cemento-enamel junction. In stage E, the root length is still less than the crown height, and in stage F, the root length is equal to or greater than the crown height. The other teeth (36–31) were also found to be significantly more undeveloped in the EPT group compared to the FT group, but with a variation between the observers (Table 3).

**Table 3:** Presentation of statistically significant differences in development stages for the teeth 37–31 when the extremely preterm group (EPT) is compared to the full-term group (FT)

Group differences EPT/FT for each individual tooth 37–31 (*p* value or NS^a^)							
Observer	37	36	35	34	33	32	31
1	0.010	NS	NS	0.002	NS	0.013	NS
2	0.000	NS	NS	NS	NS	0.002	0.005
3	0.000	0.003	0.003	NS	0.001	NS	NS
4	0.002	NS	NS	0.023	0.006	NS	NS
5	0.000	0.012	NS	NS	0.012	0.023	0.001

^a^*NS* not significant *P* ≥ 0.05)

The mean dental maturity scores within groups reported by each observer are seen in Table 4 and graphically presented in Fig. 2. The EPT group had a statistically significant lower dental maturity score than the FT group as assessed by each of the five observers (*p* ≤ 0.006).

**Table 4:** Dental maturity score within the groups extremely preterm (EPT), very preterm (VPT), and full-term (FT), as calculated from the 5 observers’ assessments of 7 left mandibular teeth (teeth 31–37) according to the method of Demirijan et al. [15]

	Mean dental maturity score (SD)			
Observer	EPT	VPT	FT	Group differences^a^, *p* values
1	86.7 (7.2)	89.1 (4.6)	91.1 (4.6)	EPT/FT 0.003EPT/VPT NSVPT/FT NS
2	81.9 (8.9)	85.2 (6.7)	88.1 (6.0)	EPT/FT 0.001EPT/VPT NSVPT/FT NS
3	83.7 (7.8)	86.4 (5.2)	89.1 (4.4)	EPT/FT 0.000EPT/VPT NSVPT/FT NS
4	84.6 (8.5)	86.7 (5.6)	89.0 (4.3)	EPT/FT 0.006EPT/VPT NSVPT/FT NS
5	82.3 (7.4)	86.6 (5.1)	88.3 (4.3)	EPT/FT 0.001EPT/VPT NSVPT/FT NS

^a^*NS* not significant

### Intra- and inter-observer agreement

Kappa values for inter-observer agreement for teeth 31–37 varied between 0.31 and 0.71, with the lowest value for tooth 33 (0.31, fair agreement) and the highest for tooth 36 (0.71, substantial agreement) (Table 5).

**Table 5:** Inter-observer agreement: kappa values for all teeth and observers

Tooth	37	36	35	34	33	32	31
Kappa value	0.50	0.71	0.44	0.42	0.31	0.44	0.44

Kappa values for intra-observer agreement for teeth 31–37 related to the observer varied between 0.16 and 1.00. If the incisors were excluded, the Kappa values varied between 0.42 and 0.93 (fair to almost perfect agreement) (Table 6).

**Table 6:** Kappa values for intra-observer agreement for teeth 31–37 related to observer

Tooth	37	36	35	34	33	32	31
Observer							
1	0.75	0.70	0.74	0.43	0.45	0.57	0.52
2	0.93	0.91	0.87	0.90	0.65	0.86	1.00
3	0.69	0.67	0.77	0.71	0.68	0.22	0.16
4	0.87	0.81	0.83	0.85	0.52	0.33	0.26
5	0.63	0.63	0.53	0.42	0.51	0.51	0.34

## Discussion

All observers assessed tooth 37 to be significant in the more undeveloped stages in the EPT group compared with the FT group (*p* ≤ 0.01). The other teeth (36–31) were also found to be significantly more undeveloped in the EPT group compared to the FT group, but with a variation between the observers. When the dental maturity scores for all 5 observers were calculated and compared for the preterm and full-term-born children, the children in the EPT group had the significantly lowest maturity score (*p* ≤ 0.006). This means that the lower the gestational age, the greater delay of tooth development at 9 years of age.

It has been reported earlier that the development of the permanent dentition differs between gender and ethnicities [19]. In order to reduce potential confounding factors like ethnic difference, only Caucasians were included in the study, and thereby, a homogenous sample was made. Another strength of the present study includes that two strictly defined groups of preterm children have been compared with a well-defined and well-matched control group of full-term children. The preterm children included in this study were included according to their gestational age, which was determined by ultrasonography. This means that no full-term child with low birthweight (small for gestational age) was included in the preterm groups.

The image quality of the panoramic radiographs was optimal, as radiography was performed under standardized conditions and repeats were made when image quality was unacceptable. This was proven by the fact that few teeth were judged as not possible to assess. Most observers reported difficulties in assessing the incisors due to superimposition of the spinal cord in the radiographs, which is a consequence of image acquisition for panoramic radiography.

To strengthen the reliability of the scoring, five observers performed the assessments and all observers duplicated 23% of the assessments. The observers were calibrated prior to the definitive assessments, and all assessments were performed without the observer being aware of to which group the panoramic radiographs belonged. The observers included in this study represent different professional experiences as well as different time lengths of expertise. If observer agreement is low or if only one observer performs the assessments, the reliability of the results can be questioned. As diagnostic accuracy efficacy is dependent on accuracy and observer performance [20], a low agreement between observations adversely affects the diagnostic accuracy of the method. The inter-observer agreement showed moderate strength but when comparing the maturity score of the different groups, a distinct and consequent pattern could be seen for all observers (Fig. 2). A few low Kappa values for two of the observers were registered for tooth 31 and 32 when intra-observer agreement was calculated, which can be explained by the difficulties to clearly observe the roots due to superimposition of the spinal cord in this region.

Many studies have indicated that prematurely born children experience significant growth failure in their early childhood [21, 22] and that compensatory growth occurs up to adolescence [21, 23]. This phase of accelerated growth constitutes the “catch-up growth” phenomenon. A follow-up study of extremely preterm children, born before 26 completed weeks of gestation, attained poor growth in their postnatal period and early childhood. This was followed by catch-up growth up to the age of 11 years, but nevertheless the children remained smaller and had significantly lower values for all the three growth parameters (length, weight, and head circumference) than the full-term born controls at 11 years [23]. These results correspond with the results from the anthropometric measurements in the present study (Table 2).

There are few studies available concerning extremely preterm children and tooth development. Due to the earlier classification of prematurity based on birth weight, even fewer studies are strictly comparable to this study. Seow et al.’s study [11] aimed to examine the influence of low birth weight on dental maturity of the permanent teeth by using the method by Demirijan et al. [15]. The material consisted of children born between gestational weeks 24 and 35. The results are to some extent similar to the results of this study and demonstrate a delay of dental maturity for the preterm children. The delay seems to be greater at a younger age and seems to increase with the decreasing birth weight. At 9 years, however, the children of Seow et al. [11] had experienced a catch-up in growth and the delay of the dental development was no longer seen, contradictory to our findings. This may be because the categorization of the children was divided by birth weight and not gestational age, which complicates a direct comparison. Backström et al. [12] also used the method by Demirijan et al. [15] and found no difference in dental maturity score for the permanent dentition when comparing preterm children born before gestational week 37 to full-term controls. This could possibly be explained by the included children being of a higher gestational age and also a little older at the examination (9–11 years old), and thus, a catch-up in growth had occurred by then. Our findings indicate that especially extremely preterm children have a delayed dental maturity at 9 years.

The general differences of dental maturity measured by a maturity score and evaluated on a group level may be explained by several factors. These include, for example, the general delay of growth in the patients in this study, as observed by a significantly shorter height, lower weight, and smaller head circumference at time of examination (Table 1). Craniofacial morphology is affected and regulated by several interacting factors including environmental, genetic, and hormonal interactions as well as supply of nutrition and epigenetic factors. Disturbances in this process may result in a change in the size and shape of the craniofacial complex [24]. Premature birth has been shown to affect the morphology of the craniofacial complex [5]. The formation of tooth 37 is initiated during the second or third year of life. At this time, the EPT children can be expected to be shorter and lighter compared to full-term children. The study from 2009 with the same study material as this study found a significantly shorter anterior cranial base and a less convex skeletal profile among EPT compared to full-term children. Additionally, EPT and VPT children had significantly shorter maxillary lengths compared to full-term children [5]. Considering these results, one can speculate that the length of the mandibular arch is not as developed among the EPT children compared to the full-term controls at age 9. Therefore, the posterior space of the mandible may be limited and interfere with the development of tooth 37, which in turn, might develop later. This can explain some of the findings in this study concerning the reported delay of tooth 37. Furthermore, tooth 37 develops later than the other teeth and has therefore less time for the so-called catch-up growth, which also could lead to the significant delay of maturity. To confirm these theories, more research is needed.

## Conclusion

The increased survival rate of the extremely preterm children in particular contributes to a new group of children in society. The findings of this study suggest a general delay in tooth development for the extremely preterm children at age 9. The dental clinician should be aware that the delay in tooth development and eruption could have an impact on the timing of orthodontic diagnostics and potential treatment.
